# Comparison of magnitude and summated vector mean of surgically induced astigmatism vector according to incision site after phakic intraocular lens implantation

**DOI:** 10.1186/s40662-021-00257-z

**Published:** 2021-09-02

**Authors:** Kazutaka Kamiya, Wakako Ando, Masahide Takahashi, Nobuyuki Shoji

**Affiliations:** 1 Visual Physiology, School of Allied Health Sciences, Kitasato University, 1-15-1 Kitasato, Minami, Sagamihara, Kanagawa 252-0373 Japan; 2 Department of Ophthalmology, School of Medicine, Kitasato University, Sagamihara, Japan

**Keywords:** Surgically induced astigmatism, Corneal astigmatism, Mean, Summated vector mean, Centroid, Vector analysis, Temporal incision, Superior incision, ICL implantation

## Abstract

**Background:**

To compare the arithmetic mean (M-SIA) and the summated vector mean of surgically induced astigmatism (SVM-SIA) according to the incision site after phakic intraocular lens (Visian implantable collamer lens (ICL), STAAR Surgical) implantation.

**Methods:**

This study comprised 121 eyes of 121 consecutive patients undergoing ICL surgery through a 3.0-mm temporal or superior clear corneal incision. The magnitude and the axis of corneal astigmatism preoperatively and 3 months postoperatively were measured using an automated keratometer. The M-SIA and the SVM-SIA were determined according to the incision site.

**Results:**

The magnitude of corneal astigmatism significantly increased from 1.23 ± 0.59 D preoperatively to 1.46 ± 0.72 D postoperatively in the temporal incision group (Wilcoxon signed-rank test, *P* < 0.001), but it significantly decreased from 1.09 ± 0.36 D preoperatively to 0.86 ± 0.41 D postoperatively in the superior incision group (*P* < 0.001). The M-SIA was 0.48 ± 0.30 D, and the SVM-SIA was 0.23 ± 0.52 D at a meridian of 82° in the temporal incision group. The M-SIA was 0.57 ± 0.30 D, and the SVM-SIA was 0.47 ± 0.45 D at a meridian of 1° in the superior incision group.

**Conclusions:**

ICL implantation induces the M-SIA by approximately 0.5 D, but the SVM-SIA decreased to 50% and 80% of the M-SIA in magnitude through temporal and superior incisions, respectively. The direction of the SVM-SIA showed a tendency toward corneal flattening to the incisional site. It should be noted that the M-SIA is somewhat different from the SVM-SIA according to the incision site.

*Trial registration* University Hospital Medical Information Network Clinical Trial Registry (000044269)

## Introduction

Posterior chamber toric phakic intraocular lens implantation (EVO Visian toric implantable collamer lens (ICL) with KS-AquaPORT; STAAR Surgical, Monrovia, CA, USA) has been broadly recognized as a safe and effective refractive procedure for the correction of moderate to high ametropia [1–8]. We currently calculate toric ICL power using the online calculation and ordering system from the ICL manufacturer based on the astigmatism decomposition method [1]. Considering that both non-toric and toric ICL surgeries require a 3.0-mm to 3.2-mm clear corneal incision, a change in astigmatism is expected in almost every eye receiving such a corneal incision. The shift in astigmatism may be strong enough to degrade visual performance and subsequent patient satisfaction since ICL implantation is one of the refractive surgeries that aim to correct spherical and cylindrical errors as much as possible. Hitherto, the software does not take surgically induced astigmatism (SIA) into account when calculating optimal ICL astigmatic power. We previously demonstrated that standard ICL implantation through a 3.0-mm temporal corneal incision induced the arithmetic mean SIA (M-SIA) by approximately 0.5 D with a with-the-rule (WTR) shift [9]. However, we only calculated the M-SIA after ICL surgery without considering the meridian of astigmatism in the study. Indeed, the M-SIA does not include information about the orientation of the astigmatic axis. The summated vector mean of SIA (SVM-SIA) consists of the magnitude and the meridian of corneal astigmatism. Hence, the SVM-SIA is considered clinically more meaningful in understanding general SIA tendencies [10, 11] and might differ from the M-SIA [12]. Moreover, ICL surgery through a superior corneal incision may be beneficial for reducing astigmatism, especially when using a non-toric ICL in WTR astigmatic eyes. To the best of our knowledge, neither studies on the SVM-SIA after ICL surgery nor those on the M-SIA through a superior incision have so far been conducted. Such studies would give us intrinsic insights into understanding the differences in the M-SIA and SVM-SIA characteristics according to the incision site in ICL-implanted eyes in daily clinical practice. The current retrospective study compared the M-SIA and SVM-SIA in eyes receiving superior and temporal corneal incisions for ICL implantation.

## Materials and methods

### Study population

The study protocol was registered with the University Hospital Medical Information Network Clinical Trial Registry (000044269). A total of 121 eyes of 121 consecutive patients (mean age ± standard deviation, 32.8 ± 7.1 years, 95% confidence interval (CI) 19.0 to 46.7 years), who underwent non-toric ICL implantation through a 3.0-mm temporal or superior clear corneal incision, and completed at least a 3-month follow-up, were enrolled in this study. This retrospective review of the clinical charts was approved by the Institutional Review Board at Kitasato University Hospital (B20-303) and followed the tenets of the Declaration of Helsinki. Written informed consent for ICL surgery was obtained from all patients after explaining the nature and possible consequences of the surgery.

## Inclusion and exclusion criteria

Inclusion criteria were 20 ≤ age < 50 years, stable refraction, moderate to high myopia and astigmatism, and no history of any trauma or ocular surgery. Exclusion criteria were eyes with concomitant corneal diseases, such as keratoconus, pellucid marginal degeneration, irregular corneal astigmatism, severe dry eye, or eyes developing intraoperative or postoperative complications. Only one eye was randomly selected for this analysis in patients who underwent bilateral ICL implantation. We mainly decided the incision site based on an ICL vault predicting formula (KS formula) according to the direction of ICL fixation (horizontal fixation through a temporal incision or vertical fixation though a superior incision) using an anterior segment optical coherence tomography device (CASIA2™, Tomey, Aichi, Japan).

### Surgical procedures

We described the details of the surgical procedures in our previous studies [9, 13]. All surgeries were performed by one experienced surgeon (K.K.) using the same technique in this study. In brief, on the day of surgery, dilating and topical anesthetic agents were applied. A model V4c or V5 ICL was implanted through a 3.0-mm temporal or superior clear corneal incision after injection of a viscosurgical substance into the anterior chamber. Next, the ICL was inserted into the posterior chamber, the viscosurgical substance was replaced with a balanced salt solution, and a miotic agent was administered. Postoperatively, steroidal (0.1% betamethasone), antibiotic (levofloxacin), diclofenac sodium medications were topically administered 4 times daily for 2 weeks, with the dose being reduced after that.

### Assessment of corneal astigmatism and surgically induced astigmatism

Preoperatively and 3 months postoperatively, we quantitatively assessed the magnitude and the axis of corneal astigmatism using an automated keratometer (TONOREFF-II, Nidek, Aichi, Japan). We measured this at least five times immediately after blinking according to the manufacturer’s instructions and used the M-SIA value for statistical analysis. We assessed the M-SIA and SVM-SIA by vector analysis, and the double angle plots for the display of the individual SIA distributions [10] by using the astigmatism double angle plot tool available on the American Society of Cataract and Refractive Surgery website (https://ascrs.org/tools/astigmatism-double-angle-plot-tool) [11]. We also evaluated the flattening effect at the meridian of incision (positive representing a flattening of that meridian and negative representing a steepening) as described previously [14].

For subgroup analysis, we also assessed the double angle plots for displaying the individual SIA distributions using unilateral data to grasp the characteristics of these SIA between the right and left eye groups.

### Statistical analysis

The normality of all data samples was first checked using the Shapiro-Wilk test. Then, since the data fulfilled the criteria for normal distribution, the paired t-test was used for statistical analysis to compare pre-and postsurgical data. Unless otherwise indicated, the results are expressed as mean ± standard deviation, and a value of *P* < 0.05 was considered statistically significant.

## Results

Table 1 shows the preoperative demographics of the study population. We found no significant differences in the preoperative biometrics, such as age (*P* = 0.284), uncorrected distance visual acuity (*P* = 0.154), corrected distance visual acuity (*P* = 0.205), manifest spherical equivalent (*P* = 0.649), manifest cylinder (*P* = 0.548), corneal astigmatism (*P* = 0.349), or mean keratometry (*P* = 0.795), between the temporal and superior incision groups. Table 2 shows the visual and refractive outcomes of ICL surgery for the temporal and superior incision patient groups.

**Table 1:** Preoperative demographics of the study population according to the incision site (temporal and superior incision groups)

	Temporal incision group	Superior incision group	*P* value
Number of eyes	80	41	–
Age (years)	32.5 ± 7.3 (range, 22 to 52)	33.5 ± 6.6 (range, 21 to 44)	0.284
UDVA (logMAR)	1.23 ± 0.27 (range, 0.40 to 2.00)	1.32 ± 0.30 (range, 0.15 to 2.00)	0.154
CDVA (logMAR)	− 0.21 ± 0.07 (range, − 0.30 to 0.00)	− 0.23 ± 0.07 (range, − 0.30 to − 0.08)	0.205
Manifest spherical equivalent (D)	− 6.46 ± 2.93 (range, − 18.25 to − 2.13)	− 6.99 ± 3.56 (range, − 16.25 to − 0.75)	0.649
Manifest cylinder (D)	− 0.61 ± 0.66 (range, − 3.25 to 0.00)	− 0.59 ± 0.37 (range, − 1.25 to 0.00)	0.548
Corneal astigmatism (D)	1.23 ± 0.59 (range, 0.00 to 3.25)	1.09 ± 0.36 (range, 0.00 to 1.75)	0.349
Type of astigmatism	With-the-rule 74 eyes (93%), against-the rule 3 eyes (4%), oblique; 2 eyes (3%), and none 1 eye (1%)	With-the-rule 39 eyes (95%), against-the rule 1 eye (2%), oblique 0 eye (0%), and none 1 eye (2%)	
Mean keratometry (mm)	43.60 ± 1.49 (range, 40.25 to 46.88)	43.55 ± 1.23 (range, 41.70 to 46.30)	0.795

*logMAR* logarithm of the minimal angle of resolution, *UDVA* uncorrected distance visual acuity, *CDVA* corrected distance visual acuity, *D* diopter

**Table 2:** Postoperative visual and refractive outcomes of ICL implantation according to the incision site (temporal and superior incision groups)

	Temporal incision group	Superior incision group	*P* value
UDVA (logMAR)	− 0.19 ± 0.10 (range, − 0.30 to 0.10)	− 0.23 ± 0.10 (range, − 0.30 to 0.10)	0.083
CDVA (logMAR)	− 0.25 ± 0.07 (range, − 0.30 to − 0.08)	− 0.28 ± 0.05 (range, − 0.30 to − 0.08)	0.567
Manifest spherical equivalent (D)	− 0.05 ± 0.23 (range, − 0.75 to 1.00)	0.05 ± 0.28 (range, − 0.88 to 0.75)	0.195
Manifest cylinder (D)	− 0.24 ± 0.31 (range, − 1.00 to 0.00)	− 0.19 ± 0.24 (range, − 0.75 to 0.00)	0.657
Corneal astigmatism (D)	1.46 ± 0.72 (range, 0.00 to 3.75)	0.86 ± 0.41 (range, 0.00 to 1.75)	< 0.001
Mean keratometry (mm)	43.67 ± 1.52 (range, 40.25 to 47.00)	43.66 ± 1.19 (range, 41.88 to 46.00)	0.972
Vault (µm)	503.5 ± 181.9 (range, 147.0 to 60.0)	437.3 ± 173.8 (range, 96.7 to 778.0)	0.332

*logMAR* logarithm of the minimal angle of resolution, *UDVA* uncorrected distance visual acuity, *CDVA* corrected distance visual acuity, *D* diopter

**Fig. 1:**
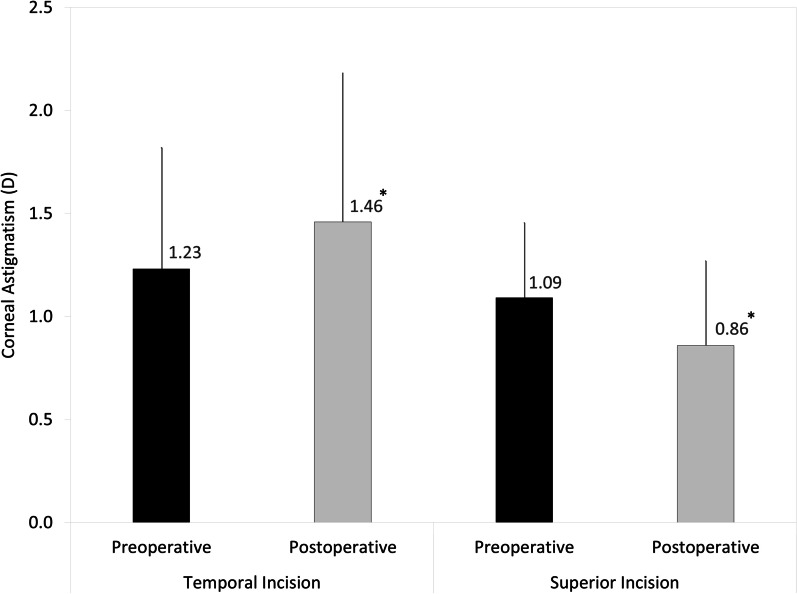
Graph showing the magnitude of corneal astigmatism preoperatively and 3 months postoperatively in the temporal and superior incision groups. The bar represents standard deviation. D, diopters. *Indicates a statistically significant difference

**Fig. 2:**
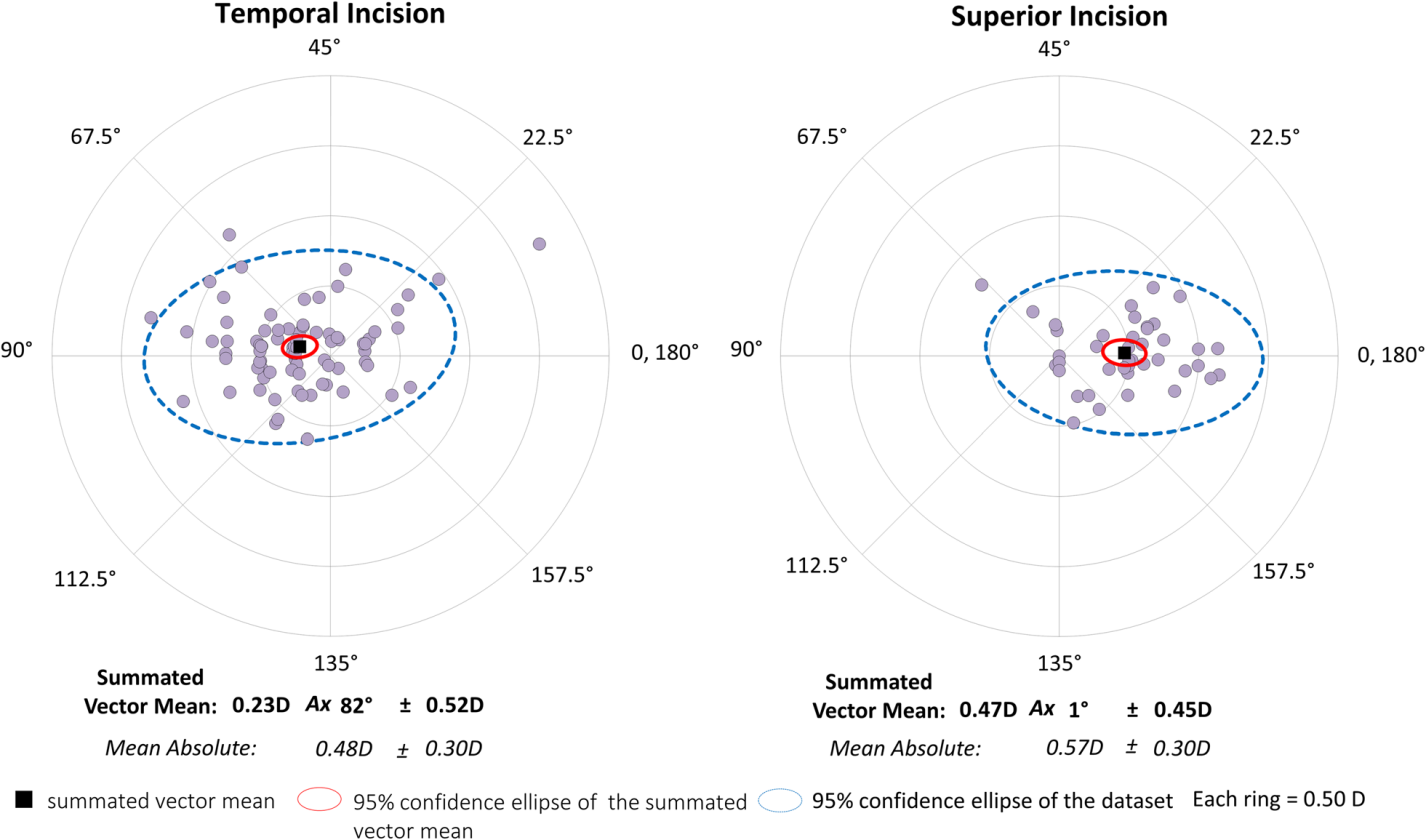
Graph showing the double angle plots of the individual surgically induced astigmatism in the temporal and superior incision groups

Figures 1 and 2 show preoperative and postoperative corneal astigmatism in magnitude and double angle plots for displaying individual SIA distributions, respectively, in the temporal and the superior incision groups. In the temporal incision group, the magnitude of corneal astigmatism was significantly increased, from 1.23 ± 0.59 diopter (D) (95% CI 0.08 to 2.38 D) preoperatively, to 1.46 ± 0.72 D (95% CI 0.05 to 2.87 D) postoperatively (Wilcoxon signed-rank test, *P* < 0.001). The M-SIA was 0.48 ± 0.30 D, and the SVM-SIA was 0.23 ± 0.52 D at a meridian of 82°. The flattening effect was 0.43 ± 0.51 D.

**Fig. 3:**
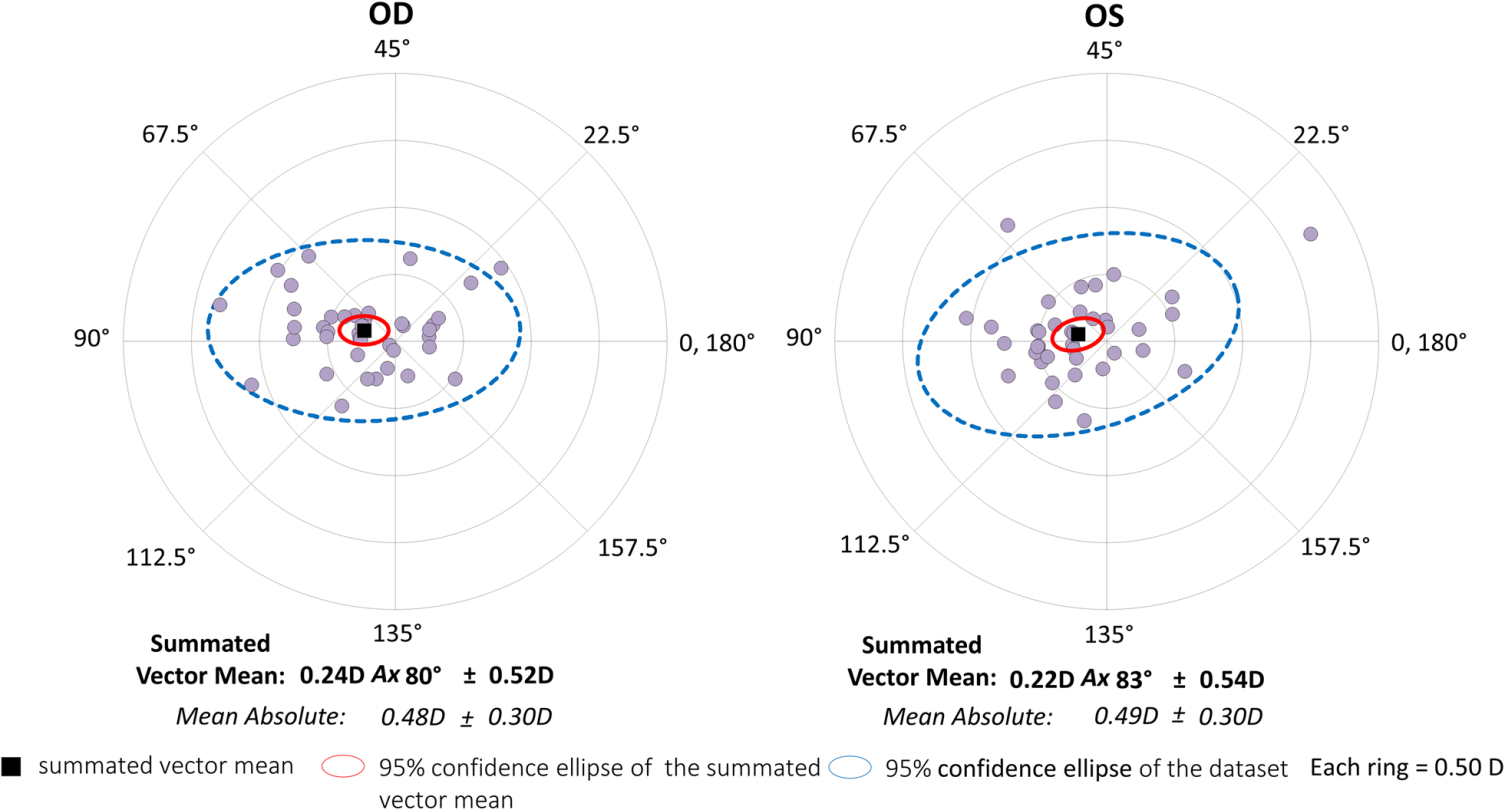
Graph showing the double angle plots of the individual surgically induced astigmatism using unilateral data in the temporal incision group

**Fig. 4:**
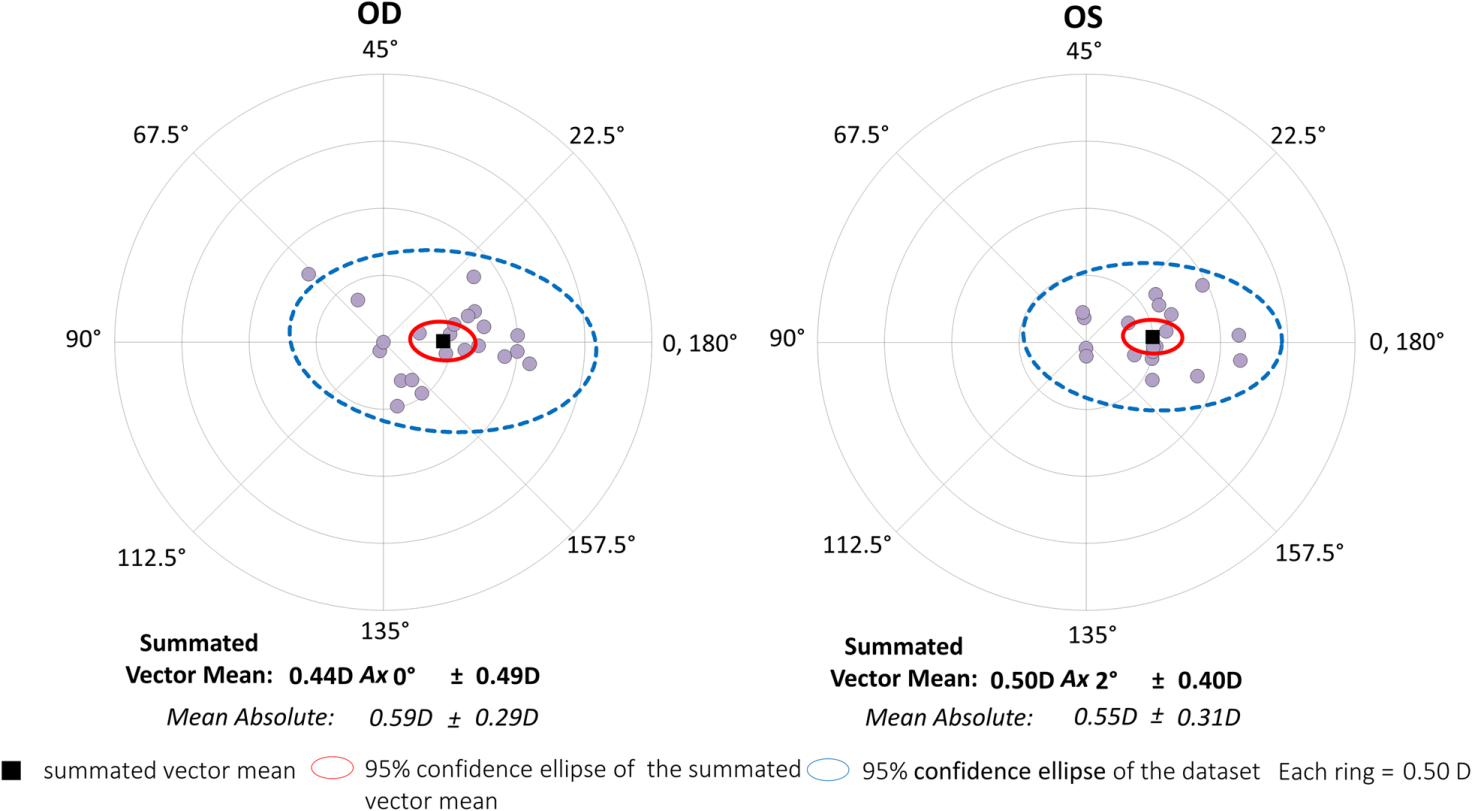
Graph showing the double angle plots of the individual surgically induced astigmatism using unilateral data in the superior incision group

In the superior incision group, the magnitude of corneal astigmatism was significantly decreased, from 1.09 ± 0.36 D (95% CI 0.3 to 1.80 D) preoperatively, to 0.86 ± 0.41 D (95% CI 0.05 to 1.66 D) postoperatively (*P* < 0.001). The M-SIA was 0.57 ± 0.30 D, and the SVM-SIA was 0.47 ± 0.45 D at a meridian of 1°. The flattening effect was 0.43 ± 0.43 D. Figures 3 and 4 show double angle plots involving individual SIA distribution of right and left eyes in the unilateral subgroups. We found no apparent differences in these SIA distributions between the two subgroups.

## Discussion

In this study, our findings showed that standard ICL surgery changed the M-SIA by approximately 0.5 D in both the temporal and superior incision groups. Our results also showed that the magnitude of corneal astigmatism significantly worsened in the temporal incision group, but significantly improved in the superior corneal incision group, possibly because most eyes had WTR astigmatism preoperatively. Our findings may also support the view that the magnitude of the SVM-SIA reduced to approximately 50% of the M-SIA in the temporal incision group, but approximately 80% of the M-SIA in the superior incision group. One possible explanation for a larger M-SIA and SVM-SIA in the superior incision group is that the distance between the incision site and the corneal center in the superior incision group is shorter than that in the temporal incision group, and thus the effect of a 3.0-mm incision in the former group becomes more prominent than that in the latter group. Although the direction of the SVM-SIA showed a tendency toward a corneal flattening in each incision location, the double angle plots of personal SIA displayed some dissimilarities in astigmatism in magnitude and direction, especially in the temporal incision group. Although the manufacturer did not reveal the toric ICL model selection and astigmatic power calculation algorithm, they are considered to be based on the astigmatism decomposition method [1]. According to our clinical results, the SVM-SIA amount was smaller than that of the M-SIA in both groups. The SVM-SIA in the superior incision group was larger than that in the temporal incision group of ICL-implanted eyes. Both SIA is small in both groups but not necessarily negligible during preoperative planning for toric ICL implantation in a clinical setting because ICL surgery is one of the refractive surgeries that aim to correct spherical and cylindrical errors as much as possible. We believe that this information may be simple but practical for both refractive surgeons and the ICL manufacturer further to optimize the astigmatic outcomes of toric ICL implantation.

To our knowledge, there have so far been no previous studies on the SVM-SIA in ICL-implanted eyes, but standard cataract surgery requiring a similar 3.0-mm temporal corneal incision has been reported to induce M-SIA by approximately 0.5 D with a WTR shift in astigmatism [15–19]. These previous findings of the M-SIA in IOL-implanted eyes were almost identical to our current results of the M-SIA in ICL-implanted eyes, but the SVM-SIA was smaller than the M-SIA in their studies by approximately 30 to 40%. We believe that the SVM-SIA has advantages over the M-SIA to understand the aggregate SIA tendencies of ICL surgery. Therefore, the SVM-SIA should be integrated into the manufacturer’s toric ICL software during preoperative planning. In addition, we should be aware that the SVM-SIA through a superior incision was somewhat different from that through a temporal incision after ICL implantation.

At present, we cannot refute the possibility that the corneal diameter played some role in SIA after ICL surgery, especially in eyes with a smaller corneal diameter. Theodoulidou et al. showed that SIA at 6 months postoperatively was 0.77 ± 0.43 D, 0.69 ± 0.34 D, 0.62 ± 0.36 D, and 0.49 ± 0.27 D, in the white to white distance of ≤ 11.6 mm, 11.7 to 11.9 mm, 12.0 to 12.2 mm, and ≥ 12.3 mm groups, respectively, indicating that the corneal diameter should always be measured preoperatively when planning cataract surgery [20]. In contrast, Zhang et al. stated that SIA was plotted against the white to white diameter and the best fit to a linear regression model with a slope of − 0.056. These findings suggest that the horizontal corneal diameter had minimal effects on SIA in uncomplicated cataract surgery through a 2.2 mm clear corneal incision [21]. A further study on the impact of the corneal diameter is still necessary to clarify this point.

Limitations of our study are as follows. Firstly, we did not measure posterior corneal astigmatism. Although the amount of posterior corneal astigmatism is far smaller than that of anterior corneal astigmatism, total corneal astigmatism using a corneal tomographer would be beneficial for understanding the precise SIA following cataract surgery [16, 17]. Secondly, being a retrospective study, background characteristics of our subjects were not controlled, and thus sampling bias may have potentially occurred. However, no significant differences in the preoperative demographics were found, and this sample size might reflect the actual demography of subjects requiring ICL surgery. Thirdly, we did not assess the astigmatic outcomes of toric ICL implantation in a clinical setting. Therefore, it still remains unclear whether these SIA may have clinical impact on the astigmatic outcomes of toric ICL implantation. Nevertheless, our results imply that it is clinically beneficial to reduce WTR corneal astigmatism when performing ICL surgery.

## Conclusion

Our results demonstrated that ICL implantation through a 3.0-mm temporal or superior corneal incision induced the M-SIA by approximately 0.5 D and that a superior incision had significantly more significant SVM-SIA reduction than a temporal incision, with a corneal flattening shifting towards the direction of the incision site. Our results also demonstrated that the SVM-SIA was somewhat different from the M-SIA in magnitude. Therefore, we believe that the SVM-SIA is still not negligible and should be applied according to the incision site, especially for selecting the appropriate toric ICL power. This information will be helpful not only for refractive surgeons during preoperative planning, but also for the ICL manufacturer to further improve the astigmatic outcomes of toric ICL surgery.

## Data Availability

The data that support the findings of this study are available from the corresponding author upon reasonable request.

## Abbreviations

ICL: Implantable collamer lens
M-SIA: Arithmetic mean of surgically induced astigmatism
SVM-SIA: Summated vector mean of surgically induced astigmatism
WTR: With-the-rule
CI: Confidence interval
D: Diopter
